# Supramolecular Biopolymer Composed of a Doubly (His)_6_‑Tagged Tandem Z‑Domain Conjugated by Zn^2**+**^ Ions

**DOI:** 10.1021/acssynbio.5c00960

**Published:** 2026-03-12

**Authors:** Syeed Ghulam Razi, Olga Krichevsky, Ellen Wachtel, Shira Albeck, Yoav Peleg, Guy Patchornik

**Affiliations:** † Department of Chemical Sciences, 42732 Ariel University, Ariel 70400, Israel; ‡ Faculty of Chemistry, Weizmann Institute of Science, Rehovot 7610001, Israel; § Life Science Core Facilities, 34976 Weizmann Institute of Science, Rehovot 7610001, Israel

**Keywords:** supramolecular-biomaterials, supramolecular-biopolymers, rationally designed materials, synthetic 2D protein assemblies, protein nanofibers, His-tagged proteins

## Abstract

Synthetic two-dimensional (2D) protein assemblies were engineered using tandem Z-domains derived from the bacterial Protein A. Assembly was induced by introducing hexa-histidine tags to both the N- and C-termini of the tandem Z-domain ((His)_6_-(Z)_2_-(His)_6_) and adding equimolar Zn^2+^ at pH 7. Two lines of evidence suggest preservation of the Z-domain’s native structure upon metal-mediated assembly: (i) far-UV circular dichroism spectroscopy; and (ii) selective binding to IgG antibodies, with no detectable interaction with IgA or IgM, consistent with the known specificity of the Z-domain. Scanning transmission electron microscopy demonstrated the formation of 2D protein assemblies exclusively in the presence of Zn^2+^ ions. The widespread use of His-tag engineering and the mild conditions required to assemble (His)_6_-(Z)_2_-(His)_6_ monomers into two-dimensional structures suggest that this approach offers a straightforward and accessible platform for the fabrication of synthetic 2D protein assemblies with potential applications in biotechnology and medicine.

## Graphical abstract

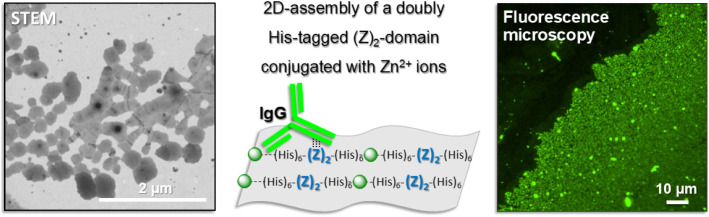

## 1 Introduction

The term “supramolecular biopolymers” describes macromolecular assemblies in which the constituent monomers do not interact covalently, but rather exploit multiple weakly attractive forces such as H-bonds, ionic bonds, van der Waals (VDW), and/or [π:π] interactions. These interactions are generally reversible. The motivation underlying the development of such biomaterials derives from experience gained with traditional biopolymers used in medicine for the replacement of damaged tissues; these materials were unable to respond to physiological cues and were therefore unable to provide the necessary dynamics of the original healthy tissue.^1^ It became clear that biomaterials must rely on reversible bonding between monomers that could, at the same time, exhibit sufficient binding affinity to preserve the stability of the artificial tissue.^2,3^ Indeed, this class of biomaterials has served in various roles in medicine (e.g., plastic surgery, vascular grafting, joint replacement, dental augmentation, bone fusion, and fixation^4–9^ ) and represents the so-called “next generation” of biomaterials. It should be emphasized that even though a given noncovalent interaction is, on its own, relatively weak, the summation of these bonds, combined with the inherent binding directionality, can generate materials with mechanical properties comparable to those of common cross-linked polymers.^10^ Perhaps the most intensively studied monomers are purpose-synthesized peptides or low molecular weight organic compounds, each possessing a hydrophobic aliphatic or an aromatic domain,^11–15^ or alternatively, urea and its analogs as conjugating entities.^16–18^ Such monomers were found to be useful for scientific and medicinal applications other than tissue replacement, which include, for example, (***i***) organ healing;^19–21^ (***ii***) drug release;^22–25^ (***iii***) scaffolds that support cell survival;^26–28^ (***iv***) biosensors;^29,30^ (***v***) enzymatic catalysts;^31^ and even (***vi***) electronic components.^32^

Perhaps the least explored interactions between monomers in supramolecular biomaterials are coordination bonds generated through the complexation of a chelator and a metal cation. Published studies have shown that chelators, such as catechol, histidine, and imidazole, can be exploited for monomer assembly when combined with a variety of relatively nontoxic cations (e.g., Zn^2+^, Fe^2+^, or Fe^3+^). However, these monomers are generally not intact proteins; rather, they are polyethylene glycol (PEG) analogs.^33–37^ Though biomaterials composed of (***i***) two protein domains,^38^ (***ii***) three different fluorescent proteins,^39^ or (***iii***) a mixture of an enzyme, a fluorescent protein, and an amyloid component^29^ are known, these successful demonstrations did not rely on [metal:chelator] complexes as the tethering moiety. It therefore appears that generating biomaterials composed of intact proteins, interacting reversibly via [metal:chelator] complexes, is an avenue that has not been adequately studied. Two recent studies can be cited, however, that do describe conjugation of His-tagged peptides or His-tagged proteins via Zn^2+^ and their use for different medicinal applications.^40,41^

This realization motivated us to develop a potentially general and simple-to-implement protocol capable of directing a protein monomer/dimer/trimer, etc., to assemble under specific conditions into a supramolecular biomaterial. Accordingly, we recently demonstrated that the introduction of a hexa-histidine (His_6_)-tag to each of the N- and C-termini of three unrelated proteins: (***i***) ubiquitin (MW ∼ 8 kDa);^42^ (***ii***) CRISPR-associated protein 9 (Cas9) (MW ∼ 163 kDa);^42^ and (***iii***) red fluorescent mCherry (MW ∼ 28 kDa)^43^ promotes the formation of fibers and/or 2D protein assemblies upon equimolar addition of Zn^2+^ (or Ni^2+^) at, or very close to, neutral pH with preservation of the protein secondary structure. Preservation of native structure upon conjugation is consistent with ***(i***) the generally nondenaturing properties of the (His)_6_-tag, thereby explaining its extensive use as an affinity ligand for protein purification since the mid-1970s;^44,45^ (***ii***) the requirement for only equimolar amounts of the cation (Zn^2+^ or Ni^2+^);^42,43^ this results in a relatively low concentration of free metal ions, which (in excess) are potentially capable of binding nonspecifically and thereby denaturing the assembling proteins; and (***iii***) the high binding affinity of the (His)_6_-tag to divalent zinc and nickel cations (Ni^2+^: *K_d_* = 0.88 μM; Zn^2+^: *K_d_* = 0.047 μM^46^).

Here, we report a novel synthetic biomaterial based on a tandem repeat of the Z-domain of Protein A, the (His)_6_-(Z)_2_-(His)_6_ domain, which binds specifically to the crystallizable fragment (Fc) of immunoglobulin G (IgG) antibodies.^47,48^ Each Z-domain adopts a triple alpha-helical bundle conformation with a hydrophobic core,^49^ conferring enhanced stability and enabling repeated use as an IgG affinity tag. Improved stability was achieved by substituting selected asparagine residues (e.g., Asn23 ^50^ and Asn28 ^51^) with threonine. To increase the potential binding capacity for IgG, we engineered a doubly hexahistidine-tagged tandem Z-domain ((His)_6_-(Z)_2_-(His)_6_) consisting of 148 amino acids (MW 16.64 kDa), with (His)_6_-tags positioned at both the N- and C-termini. We hypothesized that metal-mediated conjugation of these (His)_6_-(Z)_2_-(His)_6_ monomers would promote the formation of fibers or 2D protein assemblies capable of precipitating upon centrifugation, in either the presence or absence of bound IgG (Figure 1).

**Figure 1:**
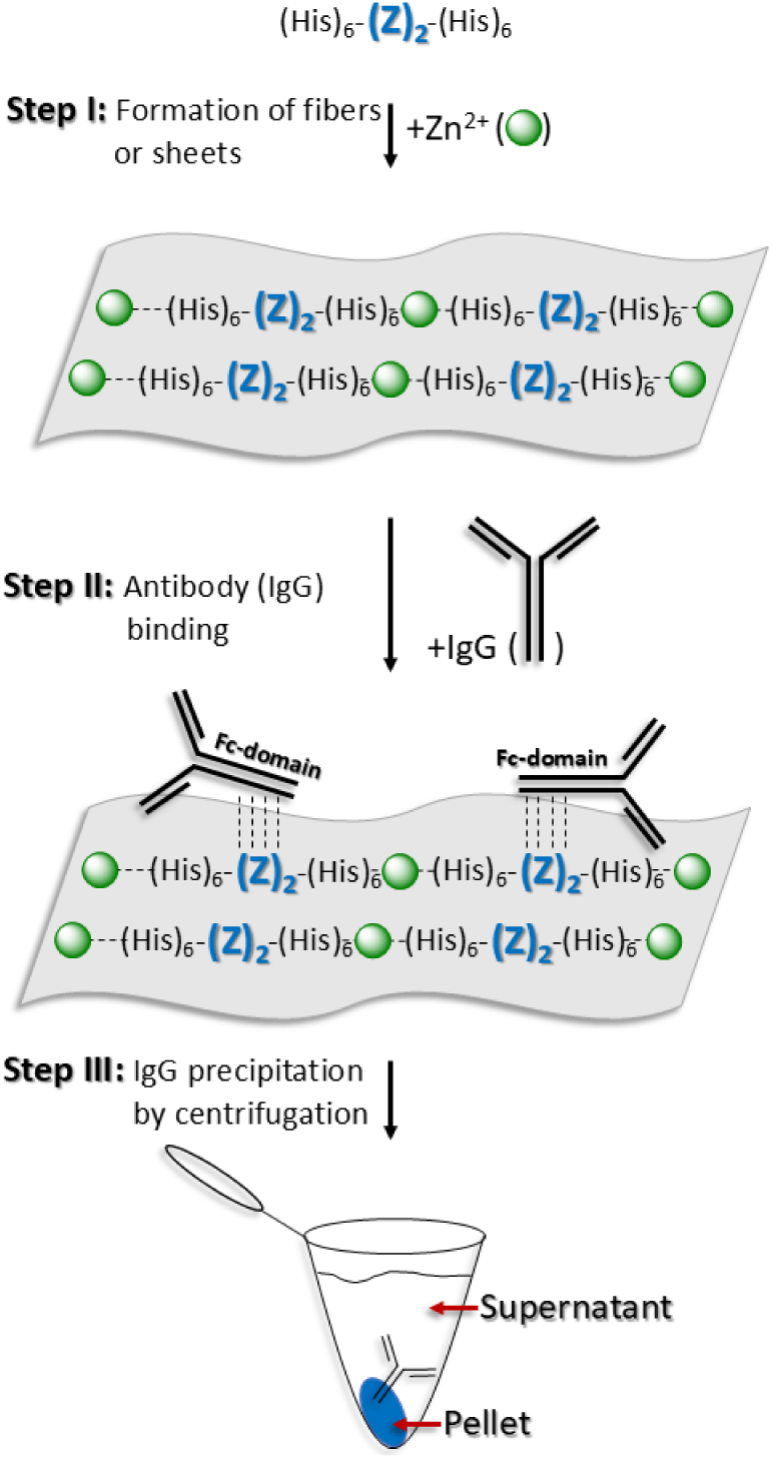
Proposed three-step protocol. Cartoon illustration of fibers or 2D protein assemblies built from a (His)_6_-(Z)_2_-(His)_6_-domain conjugated by zinc cations (**Step I**). (His)_6_-(Z)_2_-(His)_6_ fibers or 2D protein assemblies are expected to bind IgG antibodies specifically if the (His)_6_-(Z)_2_-(His)_6_ native conformation is preserved following metal conjugation (**Step II**). Centrifugation should precipitate pellets containing (His)_6_-(Z)_2_-(His)_6_-supramolecular fibers or 2D protein assemblies bound to the target IgG (**Step III**). The illustration is not drawn to scale.

The presence of IgG in the pellet would provide direct evidence for the preservation of (His)_6_-(Z)_2_-(His)_6_ specificity toward IgG antibodies. We note that Zn^2+^ cations were used for conjugation rather than Ni^2+^, which is more toxic.^52,53^ With respect to other divalent cations, additional control experiments were not conducted in this study; instead, we refer to our previous work, which demonstrated that Ca^2+^ and Mg^2+^ do not induce precipitation of a doubly His-tagged ubiquitin molecule.^42^ This behavior is likely explained, at least for Ca^2+^, by its substantially weaker affinity for the His-tag as compared to Zn^2+^ or Ni^2+^.^8^

## 2 Results

### 2.1 Gel Electrophoresis

The purity of (His)_6_-(Z)_2_-(His)_6_ was determined by SDS-PAGE under reducing conditions (Figure 2A, lane 2).

**Figure 2:**
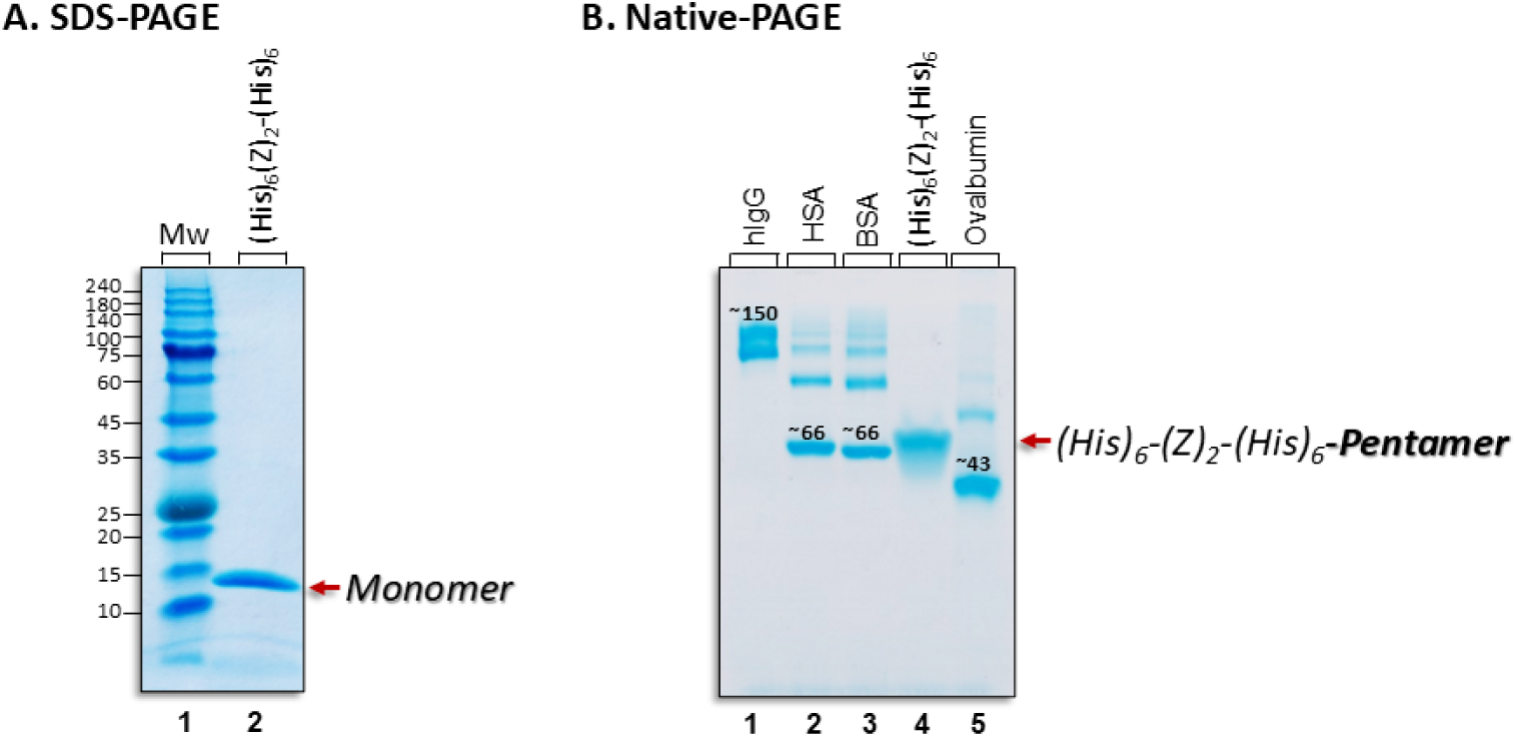
**A**. 12% SDS-PAGE analysis. Electrophoresis on a 12% SDS-PAGE gel under reducing conditions. Lane 1: Molecular weight markers (MW); Lane 2: (His)_6_-(Z)_2_-(His)_6_ (10 μg applied). **B**. 12% Native-PAGE analysis under nonreducing conditions. Lanes 1–5: Polyclonal human immunoglobulin G (hIgG), human serum albumin (HSA), bovine serum albumin (BSA), (His)_6_-(Z)_2_-(His)_6_ domain, and chicken albumin (ovalbumin). Ten μg were applied per lane. Approximate molecular weights are denoted above the four marker proteins. Gels are Coomassie stained.

A single band at ∼14.7 kDa suggested a purity level of >95% (by densitometry). The more rapid band migration of the protein, relative to its calculated MW (16.64 kDa), may be due to the presence of two (His_6_)-tags per protein monomer. Consistent with the above is the Native-PAGE gel analysis, showing a highly pure protein that migrated as a pentamer at ∼60 kDa (Figure 2B, lanes 2–5). The tendency of the protein to oligomerize was similarly observed with doubly (His_6_)-tagged red fluorescent mCherry protein; it formed dimers.^43^

### 2.2 Far-UV Circular Dichroism (CD) Spectroscopy

It was essential to determine whether the (His)_6_-(Z)_2_-(His)_6_ structure is distorted by Zn^2+^ conjugation; this was achieved via CD spectroscopy, a sensitive tool capable of determining changes in protein secondary structure.^54^ We found that the native triple-helix conformation of the (His)_6_-(Z)_2_-(His)_6_ is preserved for hours to days following the addition of zinc ions at a molar ratio of 1:1 (Figure 3).

**Figure 3:**
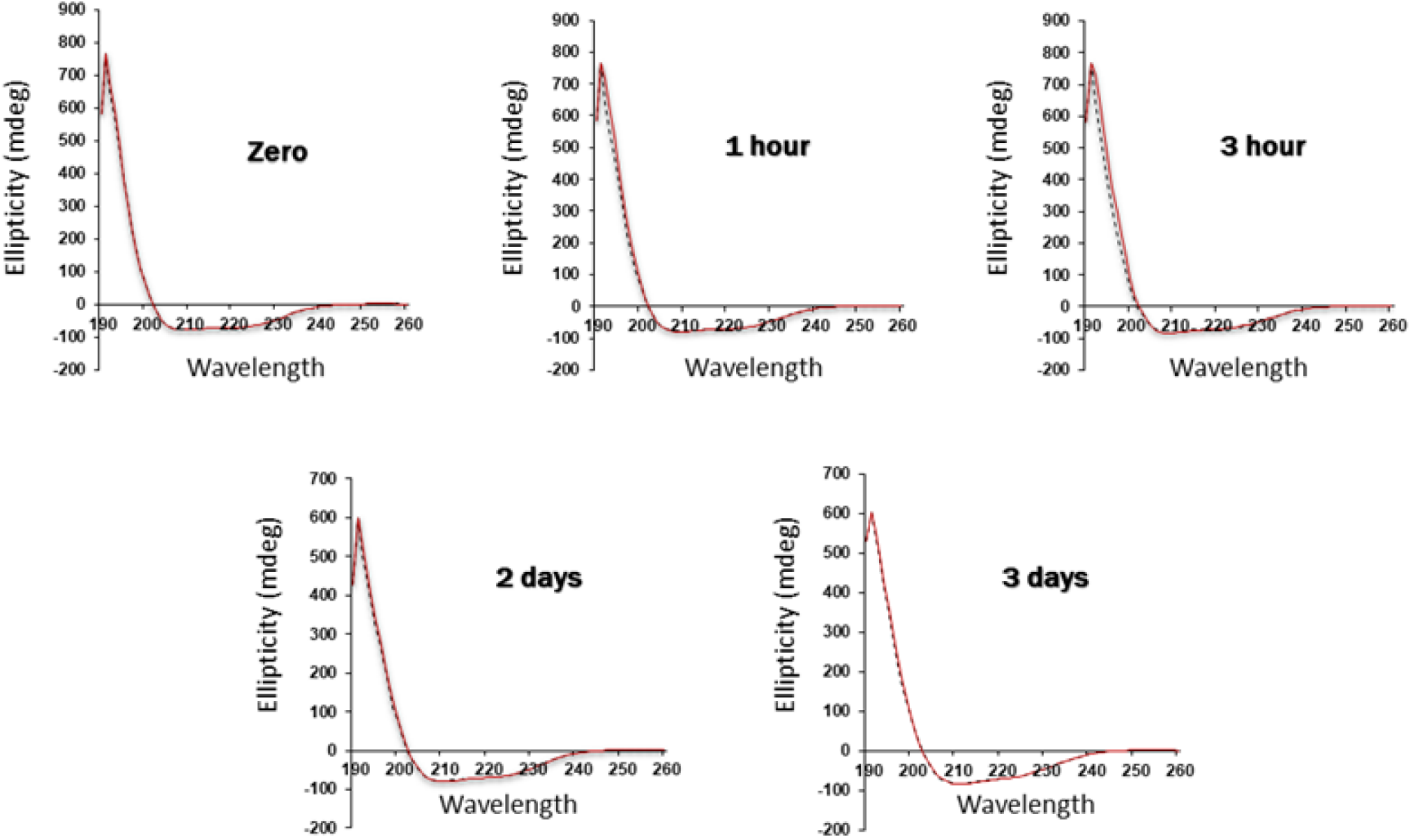
Circular dichroism (CD) spectroscopy. Far-UV circular dichroism spectra of 4.5 μM (His)_6_-(Z)_2_-(His)_6_ in the absence (black dotted line) or presence of 4.5 μM Zn^2+^ (red line) at the indicated time points. Temperature was held constant at 8 °C (281 K).

This result was encouraging, as it suggested that the affinity and specificity of the conjugated (His)_6_-(Z)_2_-(His)_6_ had been preserved and, as such, might be expected to bind to the Fc-domain of hIgG (Figure 1, Step II).

### 2.3 Scanning Transmission Electron Microscopy (STEM) Imaging of Conjugated (His)_6_-(Z)_2_-(His)_6_ Biopolymers

Evidence for the formation of fibers or 2D protein assemblies, built via the conjugation of (His)_6_-(Z)_2_-(His)_6_ monomers, was achieved with scanning transmission electron microscopy (STEM) (Figure 4). Incubation of 15 μM (His)_6_-(Z)_2_-(His)_6_ in PBS with an equimolar amount of Zn^2+^ at 25 °C (298 K) for 14 h led to the formation of 2D protein assemblies (Figure 4A–C).

**Figure 4:**
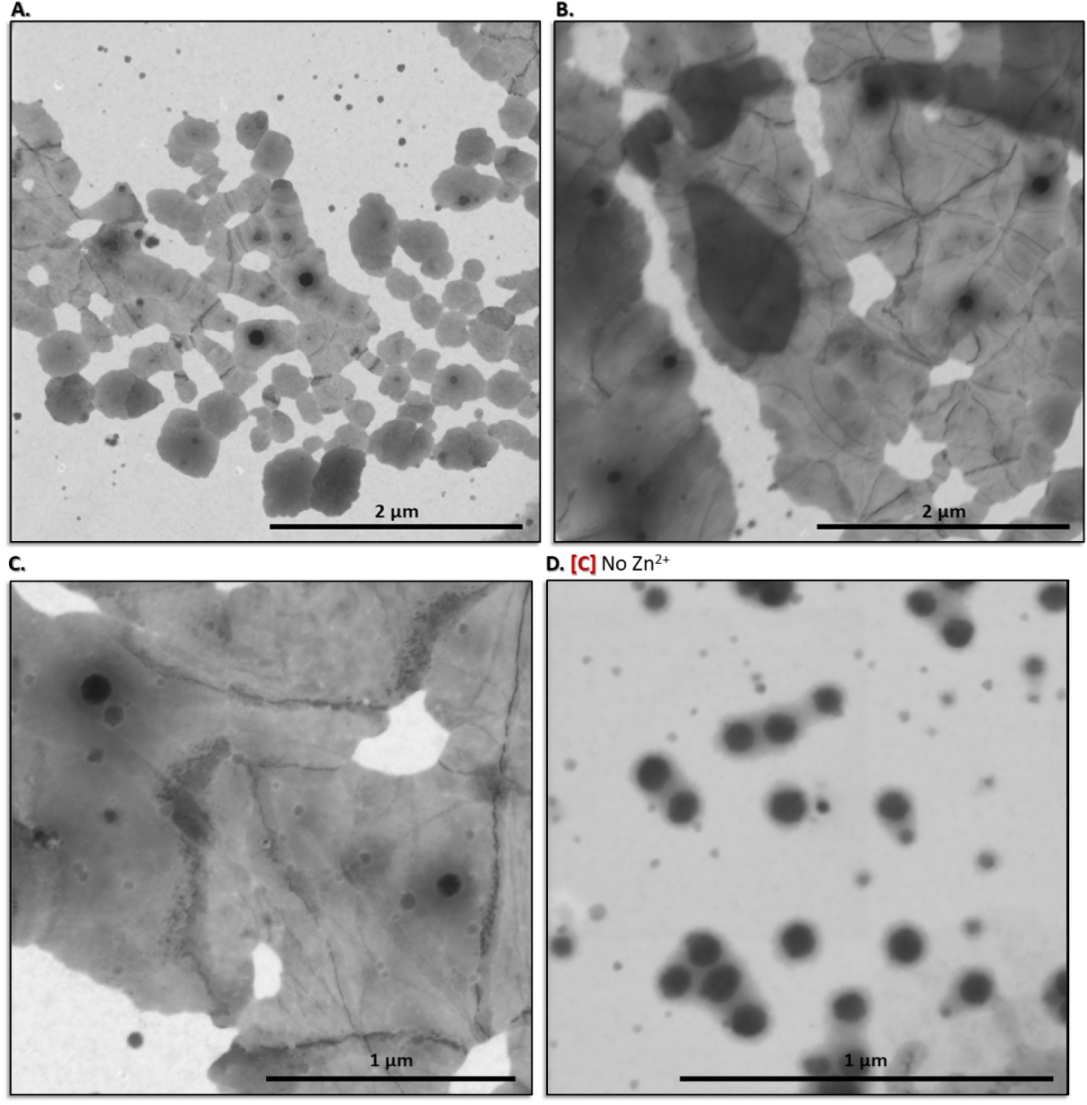
STEM imaging. **A**–**C.** Images of 15 μM (His)_6_-(Z)_2_-(His)_6_, incubated with an equimolar concentration of ZnCl_2_ in PBS at 25 °C (298 K) for 14 h. **D.** Control: as in **A–C,** but in the absence of ZnCl_2_.

Even though isolated 40–200 nm^2^ 2D protein assemblies were clearly observed, a majority appeared to be fused to each other, thereby generating layers that extended over multimicron areas (Figure 4A–C). At higher magnification, dark borderlines within the micrometer-sized 2D protein assemblies were also observed (Figure 4C). These borderlines may represent the fusion boundary between smaller tandem Z 2D protein assemblies or a (His)_6_-(Z)_2_-(His)_6_ fiber embedded within the (His)_6_-(Z)_2_-(His)_6_ 2D protein assembly. Currently, we cannot differentiate between these options, and, of course, there may be others. The mandatory dependence on Zn^2+^ ions was demonstrated by repeating the same protocol in the absence of the metal (Figure 4D). Under these conditions, only electron-dense, independent disk-like structures were visualized (Figure 4D), a few of which were observed as well in the presence of Zn^2+^ (Figure 4A–C). This finding suggests that equimolar amounts of metal were insufficient for conjugating the entire (His)_6_-(Z)_2_-(His)_6_ population, and some fraction of protein “monomers” remained free in solution.

### 2.4 Gel Electrophoresis under Reducing Conditions of hIgG Bound to Conjugated (His)_6_-(Z)_2_-(His)_6_ Biopolymers

Following the demonstration of the participation of Zn^2+^ in protein assembly, we aimed to assess whether the resulting (His)_6_-(Z)_2_-(His)_6_ 2D protein assemblies preserved their known ability to bind to the fragment crystallizable domain (Fc-domain) of IgG^48^ (Figure 1, Step II). This would provide evidence for the preservation of the native structure of the (His)_6_-(Z)_2_-(His)_6_-domains upon metal conjugation. Polyclonal human IgG (hIgG) served as the target protein and was incubated with preformed 2D protein assemblies in PBS (Figure 1, Step II). A 10 min incubation at 10 °C (283 K), followed by brief centrifugation (Eppendorf, 21130 × *g* RCF, 5 min, at 10 °C (283 K)), demonstrated that ∼67% of the hIgG population and ∼62% of the (His)_6_-(Z)_2_-(His)_6_ biopolymers (by densitometry) were not present in the supernatant (Figure 5, lane 4) relative to the total amounts added (Figure 5A, lanes 2–3) and were found in the pellet (Figure 5A, lane 6).

**Figure 5:**
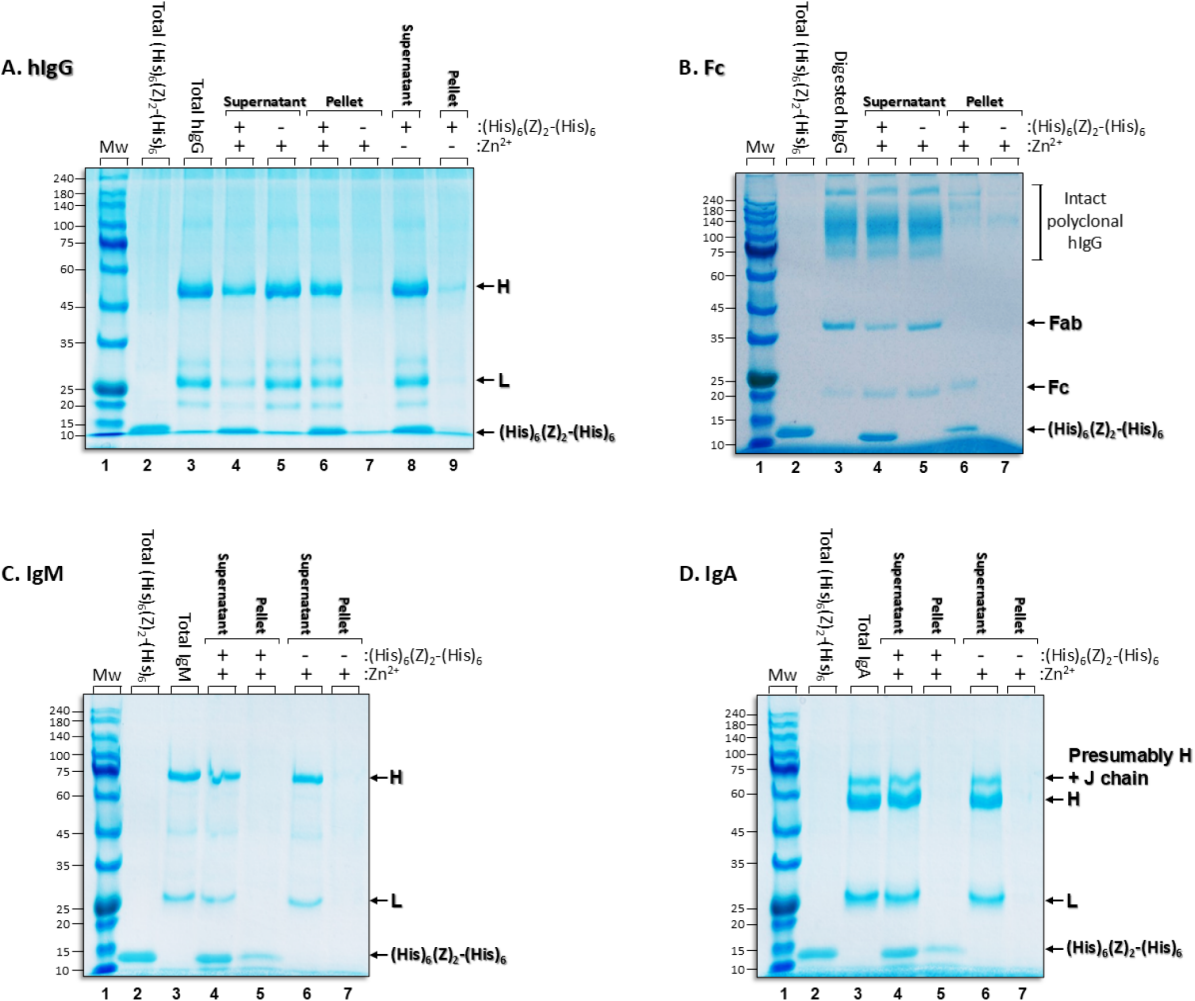
**A.** SDS-PAGE electrophoresis. **A.** Human IgG (hIgG) (run under reducing conditions) present in the supernatant or pellet following incubation with (His)_6_-(Z)_2_-(His)_6_:Zn^2+^ conjugated biopolymers. Lane 1: Molecular weight markers; Lane 2: total amount of (His)_6_-(Z)_2_-(His)_6_ applied (3.78 μg = 0.8 μM); Lane 3: total amount of polyclonal hIgG applied (11.25 μg = 0.075 μM); Lane 4: Supernatant composition following 10 min incubation at 10 °C (283 K) in PBS of hIgG with conjugated (His)_6_-(Z)_2_-(His)_6_:Zn^2+^ 2D protein assemblies; Lane 5: as in lane 4, but in the absence of (His)_6_-(Z)_2_-(His)_6_; Lanes 6–7: Pellet composition, as described for lanes 4–5; Lanes 8–9: Supernatant and pellet composition, respectively, as described for lane 4 but in the absence of (His)_6_-(Z)_2_-(His)_6_. **H** and **L** in lane 3 point to the migration of the reduced heavy (H) and light (L) chains of the hIgG population. **B.** As in **A**, but with papain-digested hIgG run under nonreducing conditions. **C,D.** As in **A**, but with 6 μg of human IgM or human IgA applied in PBS. Experiments were conducted in at least duplicate and on different days. All gels are Coomassie stained.

A control experiment in the absence of the (His)_6_-(Z)_2_-(His)_6_ resulted in ∼94% of the hIgG being in the supernatant (Figure 5A, lane 5); only trace amounts of the antibody were in the pellet (∼6%) (Figure 5A, lane 7). Very similar results were observed when the protocol was repeated without the metal (Figure 5A, lanes 8–9). The two control experiments point to the participation of the (His)_6_-(Z)_2_-(His)_6_ and Zn^2+^ ions during hIgG capture and precipitation. In the absence of the (His)_6_-(Z)_2_-(His)_6_ domain, no specific binding to IgG is observed, and in the absence of Zn^2+^ ions, micron-sized (His)_6_-(Z)_2_-(His)_6_ 2D protein assemblies—capable of being precipitated at the relatively low centrifugal force of 21130 × g—are not generated. Even though ∼10-fold higher concentration of the (His)_6_-(Z)_2_-(His)_6_ was present relative to hIgG, i.e., 0.8 μM vs. 0.075 μM, respectively, this molar ratio was not sufficient to quantitatively precipitate the entire antibody population. The water solubility of (His)_6_-(Z)_2_-(His)_6_ 2D protein assemblies may be responsible for the precipitation of only 62–57% of the total amount of (His)_6_-(Z)_2_-(His)_6_ added to the system (Figure 5A, lanes 4 and 6 vs 2).

## 3 Discussion

Trials aimed at identifying the minimum concentration of Zn^2+^ required for hIgG precipitation indicated that 1 mM represents a lower bound (data not shown). This Zn^2+^ concentration is orders of magnitude higher than the hIgG concentration used (0.075 μM) and could potentially promote nonspecific precipitation through ionic cross-linking. This was not observed; ∼96% of hIgG remained in the supernatant in the presence of 1 mM Zn^2+^ (Figure 5A, lanes 8–9 versus lane 3), indicating that Zn^2+^ alone does not induce antibody precipitation under these conditions. Evidence for the specific binding of (His)_6_-(Z)_2_-(His)_6_ to the Fc region of hIgG, rather than to the Fab region, was demonstrated using SDS-PAGE analysis of papain-cleaved polyclonal human IgG. Papain digestion yields F­(ab’)_2_ and Fc fragments, which were subsequently incubated with conjugated (His)_6_-(Z)_2_-(His)_6_:Zn^2+^ biopolymers (Figure 5B, lanes 6–7). Further evidence supporting preservation of Fc specificity was obtained by repeating the protocol with human IgA, a covalent dimer (Figure 5C), and human IgM, a covalent pentamer of hIgG (Figure 5D). The known greater binding affinity of the native Z-domain for the Fc-domain of IgG’s (*K_d_* = 10 nM),^48^ in comparison to that of IgA (3000–500 nM)^55^ or IgM (not detectable)^55^ is evident.

Upon Zn^2+^-mediated conjugation, (His)_6_-(Z)_2_-(His)_6_ 2D protein assemblies were capable of binding and potentially concentrating fluorescently labeled IgG antibodies. Commercial fluorescein isothiocyanate (FITC)-labeled IgG was added to PBS containing preformed (His)_6_-(Z)_2_-(His)_6_ 2D protein assemblies, and its spatial distribution was monitored by fluorescence microscopy (Figure 6).

**Figure 6:**
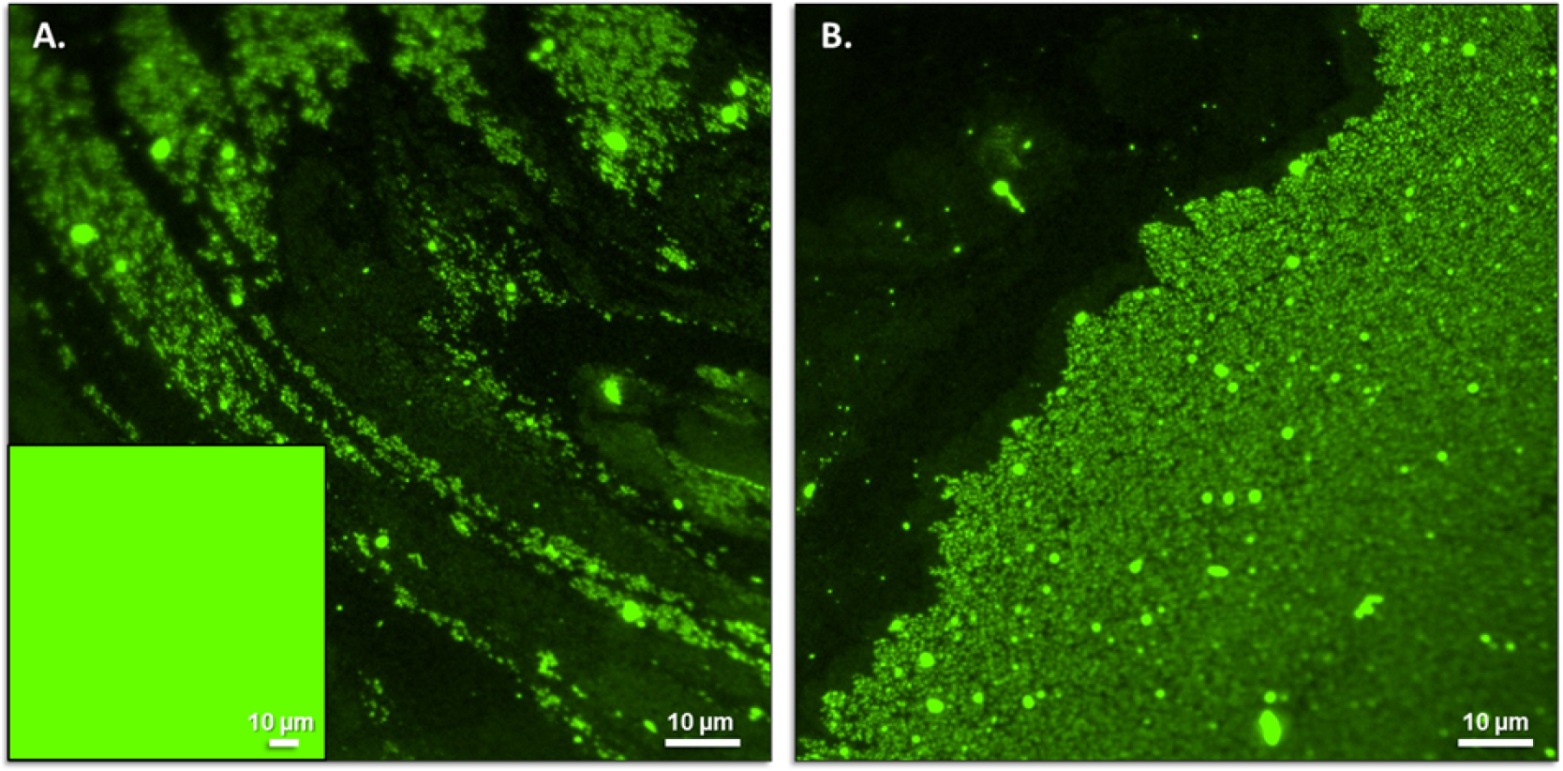
Fluorescence imaging of adsorption of fluorescent IgG to (His)_6_-(Z)_2_-(His)_6_:Zn^2+^ supramolecular polymeric 2D protein assemblies. **A**. Imaging of fluorescent antibody made possible by its specific adsorption to nonfluorescent (His)_6_-(Z)_2_-(His)_6_ 2D protein assemblies observed following overnight incubation at 8 °C (281 K) of the commercial FITC—labeled antihuman IgG: (His)_6_-(Z)_2_-(His)_6_:Zn^2+^ at a molar ratio of 1:10:13, respectively. **Inset** in **A**: in the absence of Zn^2+^. **B.** The same sample as in panel **A**, except imaging was performed at a different location on the substrate.

Following overnight incubation at 8 °C (281 K) in the presence of (His)_6_-(Z)_2_-(His)_6_, 2D protein assemblies led to the appearance of strongly fluorescent condensates (1–4 μm^2^) that were either scattered (Figure 6A) or more condensed with a lengthy, sharp boundary (Figure 6B). These results were obtained when (His)_6_-(Z)_2_-(His)_6_ was present at a 10x molar excess over the fluorescent IgG. These observations suggest that the [IgG:(His)_6_-(Z)_2_-(His)_6_-domain] molar ratio influences supramolecular assembly, although the underlying mechanism remains unclear. The high affinity of Zn^2+^ for the (His)_6_-tag (*K_d_* = 0.047 μM^46^) combined with the strong affinity of the Z-domain to the Fc-domain of IgG’s (*K_d_* = 10 nM^48^) may provide at least a partial answer. It was encouraging to find that only a small molar excess of Zn^2+^ (10–30%) over that of the (His)_6_-(Z)_2_-(His)_6_ domain was required to promote assembly, allowing Zn^2+^ concentrations to be maintained at low micromolar levels (50 μM). At these concentrations, protein denaturation due to free Zn^2+^ is unlikely. Process dependence on (His)_6_-(Z)_2_-(His)_6_ 2D protein assemblies was demonstrated by repeating the experiment in the absence of both the tandem Z-domain monomer and Zn^2+^, which led to a homogeneous distribution of the fluorescent antibody (Figure 6A, inset). IgG adsorption to the (His)_6_-(Z)_2_-(His)_6_ 2D protein assemblies appears to be a rapid process, as fluorescent supramolecular aggregates were observed even when incubation times were reduced from overnight to 20 min (data not shown).

The production of 2D conjugated protein assemblies built from His_6_-(Z_2_)-(His_6_) domains is consistent with our earlier reports showing similar 2D protein assemblies obtained from doubly (His)_6_-tagged ubiquitin,^42^ Cas9,^42^ and mCherry.^43^ A schematic illustration (Figure 7) demonstrates how 2D protein assembly is made possible via three or four (His)_6_-tags forming a complex with a Zn^2+^ cation. The model is based on literature reports^56^ that Zn^2+^ forms tetrahedral coordination bonds in zinc-finger proteins, while in aqueous media, Zn^2+^ is coordinated in octahedral geometry to water molecules^57^ and (His)_6_-tagged proteins.^58^

**Figure 7:**
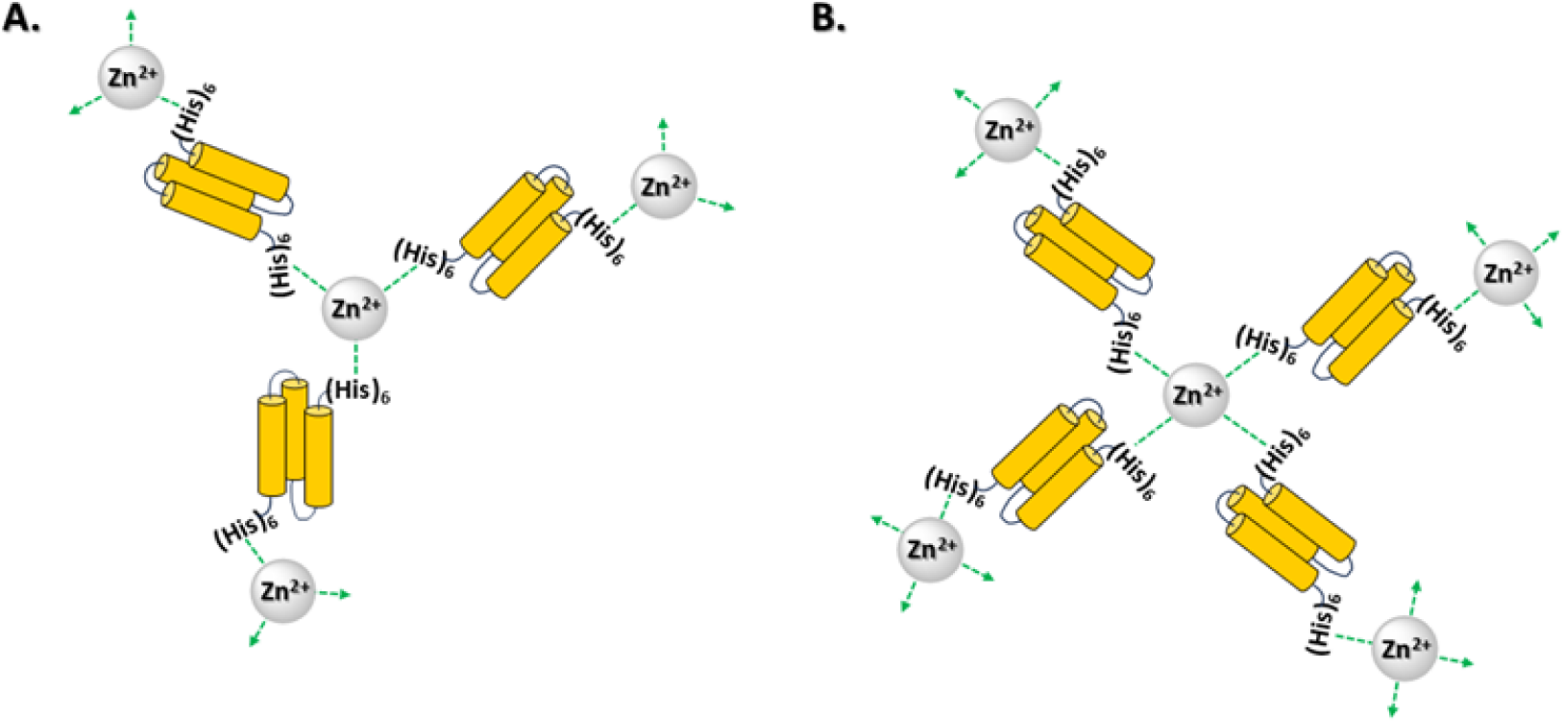
Schematic illustration suggesting formation of 2D protein assemblies built from tandem Z-domains when three (His)_6_-tags (A) or four (His)_6_-tags (B) participate in metal coordination with zinc cations. For clarity, each tandem Z domain is drawn as a single —rather than two—triple-helix bundle. The illustration is not to scale.

We also note that 2D protein assemblies could potentially be prepared from more than one doubly His-tagged protein. For example, 2D protein assemblies built from a tandem Z-domain and an enzyme (e.g., horseradish peroxidase, HRP) could improve the sensitivity of direct enzyme-linked immunosorbent assay (ELISA) assays, as illustrated in Figure 8. The tandem Z-domain is expected to bind to the Fc-domain of the primary antibody bound to the target antigen, while multiple copies of HRP would generate a more intense signal (Figure 8 **B**) than traditional direct ELISA assays (Figure 8 **A**).

**Figure 8:**
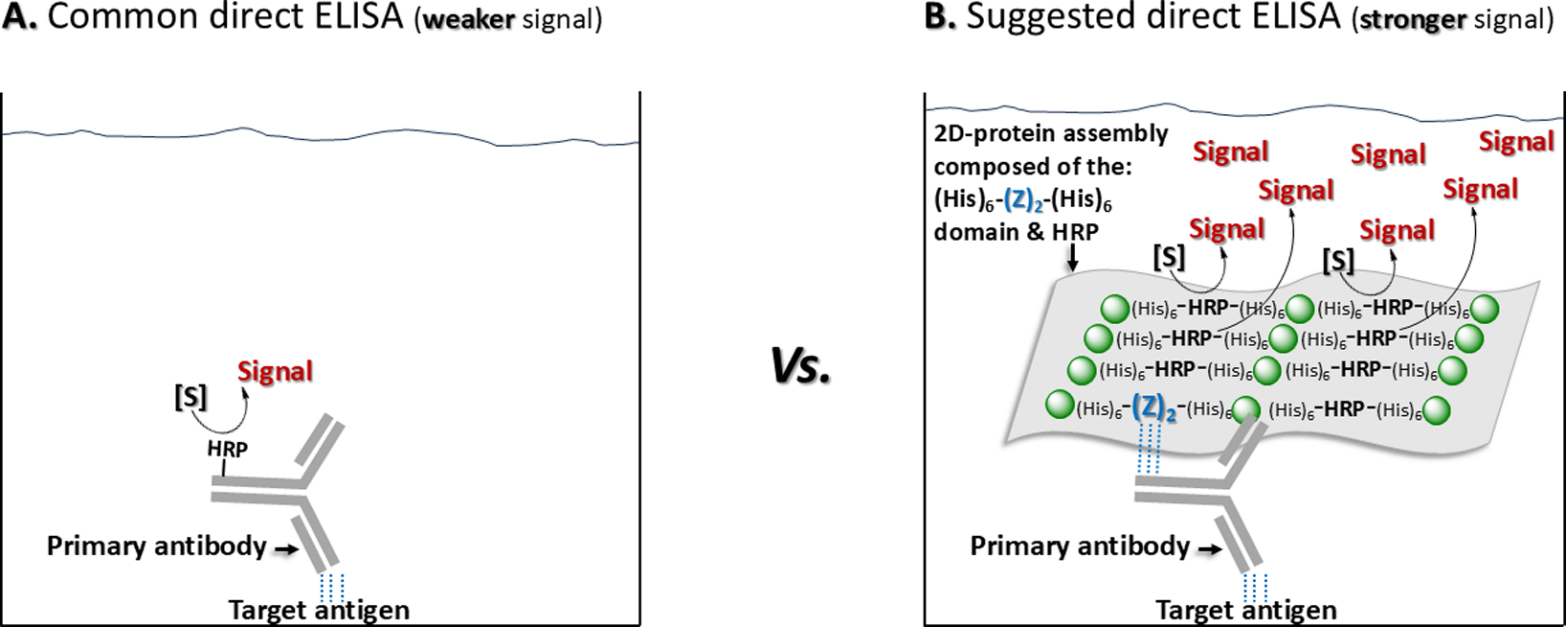
Schematic illustration showing how direct ELISA assays might benefit from greater sensitivity with the use of 2D protein assemblies built from the horseradish peroxidase enzyme (HRP) conjugated via zinc cations (green spheres) to the [His_6_)]_2_-Z_2_ domain. The binding of the latter to the Fc-domain of a primary antibody bound to the antigen of interest would direct a large number of HRPs to the antigen, which in turn would produce an intense signal due to the large copy number of HRPs on site. [S], substrate.

## 4 Conclusions

This study demonstrates that hexa-histidine tags, widely employed in affinity chromatography, can also serve as effective ligands for controlled protein assembly when placed at both the N- and C-termini of a protein of interest. Because this modification poses a minimal technical challenge, this approach should be readily generalizable. The high affinity of Zn^2+^ for His_6_-tags (*K_d_* = 0.047 μM^46^) enables assembly at low metal concentrations (e.g., 50 μM), facilitating the formation of protein fibers or 2D protein assemblies under mild conditions. We suggest that, in general, doubly (His)_6_-tagged proteins can function as modular, monomeric “building blocks” for synthetic protein assemblies, with the (His)_6_-(Z)_2_-(His)_6_ domain representing the fourth such example, following ubiquitin,^42^ Cas9,^42^ and red fluorescent mCherry.^43^ Although the number of studies in the literature in which His-tags have been used for the preparation of biomaterials and/or nanomaterials is quite limited, a few such reports, in addition to ours, have recently been published.^40,41^ We do believe, however, that our work presents fundamentally different capabilities, due to our introduction of a protocol for the preparation of micron-sized, metal-coordinated, two-dimensional protein assemblies rather than spherical nanoparticles (as in Laguna et al.^41^) or peptide-based nanowires (as in Clegg et al.^40^). The straightforward biochemistry with which 2D protein assemblies can be prepared may lead to a solution for applications in which protein layers would be preferred over 1D protein fibers or protein beads—for example, applications in which coating a surface with a thin layer of functional protein would provide a technological/pharmaceutical advantage over other possible geometries.

## 5 Materials and Methods

### 5.1 Materials

Reagent-grade sodium chloride (NaCl), magnesium chloride (MgCl_2_), zinc chloride (ZnCl_2_), sodium dodecyl sulfate (SDS), tris-HCl, isopropyl beta-d-1-thiogalactopyranoside (IPTG), phenylmethylsulfonyl fluoride (PMSF), IgA, IgM, and bovine serum albumin (BSA) were all purchased from Sigma-Aldrich, Israel. The IgG fragmentation kit, FragIT, was purchased from Genovis. pET28a, a plasmid containing the ZZ-domain gene, was purchased from Novagen Merck, Germany. BL21­(DE3), the cell type in which the ZZ-domain was expressed in *E. coli*, was purchased from Thermo Fisher Scientific, USA. DNase was purchased from Invitrogen, Israel. The source of the Z_2_-domain variant (6YLM, MW = 30.58 kDa) is the Discom asp. mushroom. Commercial antihuman FITC-labeled whole IgG antibody, expressed in goat, was purchased from Sigma-Aldrich. Polyclonal human immunoglobulin G (IgG) (∼150 kDa) (Lee Biosolutions, 340–21, purity ≥ 95%) was used. Bovine serum albumin (BSA) (∼66.4–66.5 kDa) (Merck, A8806, purity ≥ 96%) was also used. Human serum albumin (HSA) (66.5–66.7 kDa) (Merck, A5843, purity ≥ 96%) and chicken serum albumin (ovalbumin) (42.7 kDa) (Merck, A5503, purity ≥ 98%) were included. Polyclonal IgA from human colostrum (high purity ≥ 95% by HPLC) was purchased from Sigma, I2636. Polyclonal IgM derived from bovine serum and exhibited high purity (≥95% by HPLC, Sigma, I8135).
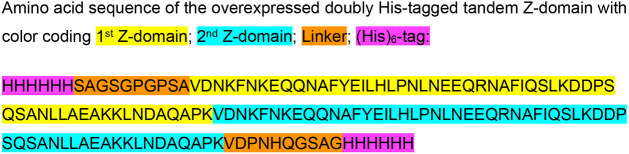


### 5.2 Methods

#### 5.2.1 Cloning, Expression, and Purification of the (His)_6_-(Z)_2_-(His)_6_ Domain with Double Hexa-His Tags

The tandem Z-domain was amplified from plasmid pBG1805, obtained from an internal collection in the Weizmann Institute of Science bacteriology unit. Primers 28ZZ_F 5′- CAGCCATCATCATCATCATCACTCCGCGGGTTCCGGTCCTGGACCATCGGCC) and 28ZZ_R­(5′- CTTAGTGGTGGTGGTGGTGGTGACCCGCGGAACCCTGATGATTCGGGTCTAC) were used for PCR amplification. Cloning was performed by the Restriction-Free (RF) method,^54^ replacing human ubiquitin in plasmid pET28-UB-(His_6_)_2_.^40^ The newly generated plasmid pET28-tandemZ-domain-(His_6_)_2_ contains an N-terminal hexa-histidine tag followed by a flexible linker (HHHHHHSAGSGPGPSA) and an additional C-terminal hexa-histidine tag appended via a linker sequence (VDPNHQGSAGHHHHHH), thereby encoding a doubly tagged protein. Both constructs were expressed in *E. coli* BL21­(DE3) cells grown in lysogeny broth (LB) medium supplemented with 30 μg/mL kanamycin. A 5-L culture was induced at OD_600_ = 0.6–0.8 with 200 μM isopropyl β-d-1-thiogalactopyranoside (IPTG) and incubated for 16–18 h at 15 °C. Cells were harvested and lysed using a cooled cell disruptor (Constant Systems) in lysis buffer composed of PBS with 20 mM imidazole, supplemented with lysozyme (200 KU/100 mL), DNase I (20 μg/mL), 1 mM MgCl_2_, 1 mM PMSF, and a protease inhibitor cocktail. Following centrifugation to remove debris, the supernatant was loaded onto a 5 mL HisTrap FF column (Cytiva), extensively washed, and eluted with buffer containing 0.5 M imidazole. Eluted fractions were pooled and dialyzed for 16–18 h at 4 °C against PBS, flash-frozen in aliquots using liquid nitrogen, and stored at −80 °C.

#### 5.2.2 Gel Electrophoresis: SDS-PAGE and Native-PAGE

Samples (0.2 μg) containing the expressed and purified (His_6_)-Z_2_-(His_6_) domain were analyzed on SDS–PAGE using a 12% resolving gel prepared according to the protocol of Laemmli et al.,^59^ under reducing conditions. Samples containing the expressed and purified (His)_6_-(Z)_2_-(His)_6_ domain were also analyzed on a 12% Native-PAGE gel under nonreducing conditions according to the protocol of Trudel et al.^60^ Accordingly, 12% gels were prepared by using an acrylamide/bis-acrylamide mixture (29:1; Bio-Lab) devoid of sodium dodecyl sulfate (SDS) and beta-mercaptoethanol (as a reducing agent). While 12% “resolving gels” were composed of 375 mM Tris at pH 8.8, the 4% “stacking gels” contained 125 mM Tris at pH 6.8. Polymerization was initiated by the addition of 0.05% ammonium persulfate (APS), followed by 0.05% *N*,*N*,*N*′,*N*′-tetramethylethylenediamine (TEMED). Protein samples were prepared in a native sample buffer containing 62.5 mM Tris (pH 6.8), 10% glycerol, and 0.01% bromophenol blue. No reducing agent or denaturant was added. Samples were not heat-treated prior to loading onto the gel. Electrophoresis was carried out at 4 °C in a Tris–glycine running buffer, i.e., 25 mM Tris and 192 mM glycine at pH ∼8.3, under a constant voltage of 80 V until the dye reached the gel front. Protein bands were stained with Coomassie Brilliant Blue R-250. A BioRad Mini-PROTEAN Tetra Vertical Electrophoresis Cell (BioRad) was used for protein separation. Molecular weight markers running under native conditions were: polyclonal human immunoglobulin G (IgG) (∼150 kDa) (Lee Biosolutions, 340–21, purity ≥ 95%), bovine serum albumin (BSA) (∼66.4–66.5 kDa) (Merck, A8806, purity ≥ 96%), human serum albumin (HSA) (66.5–66.7 kDa) (Merck, A5843, purity ≥ 96%), and chicken serum albumin (ovalbumin) (42.7 kDa) (Merck, A5503, purity ≥ 98%).

#### 5.2.3 Densitometry

The intensity of bands due to hIgG or the [(His)_6_]_2_-tandem Z domain biopolymers present in Coomassie-stained gels was quantified using ImageJ (NIH). The precipitation yield of these proteins was calculated by comparing the total amount added to each of the precipitation trials to the corresponding band intensity in the supernatant after incubation with the tandem-Z biopolymers and following a short spin.

#### 5.2.4 Far-UV Circular Dichroism (CD) Spectroscopy

(His)_6_-(Z)_2_-(His)_6_, concentration 0.07 mg/mL, was diluted in phosphate-buffered saline (PBS) and subjected to CD spectroscopy using a Chirascan CD spectrometer (Applied Photophysics). CD spectra report ellipticity (θ), proportional to the difference in absorbance of left- and right-circularly polarized light [θ = 3300° (*A*_L_ – *A*_R_)] as a function of wavelength. A quartz cell of path length 0.1 cm was used for the measurements. CD spectra were recorded with 2 nm bandwidth resolution in 1 nm steps at 25 °C (298 K) and were corrected for baseline distortion by subtracting a reference spectrum of the corresponding buffer solution.

#### 5.2.5 Scanning Transmission Electron Microscopy (STEM)

Samples containing 0.2 mg/mL (15 μM) of (His)_6_-(Z)_2_-(His)_6_ in the absence of divalent metal ions ([C]) or in the presence of 15 μM Zn^2+^ were dispersed in double-distilled water (DDW). These were loaded onto a carbon-coated copper grid Type-B (200 mesh) and dried for 16–18 h in a desiccator at 25 °C (298 K). Images were obtained using the UHR-MAIA3 TESCAN SEM with a STEM detector 420 operating at HV 25 kV. Measurements were made in duplicate for each sample and on different days.

#### 5.2.6 Binding and Precipitation of Human IgG (hIgG) or hIgG Fc-Domain

(**i**) Into 33 μL of PBS, 2 μL of 400 μM (6.3 mg/mL) (His)_6_-(Z)_2_-(His)_6_ and 10 μL of 5 mM ZnCl_2_ were added, and the mixture was incubated for 10 min at 25 °C (298 K). This was followed by the addition of 5 μL of 50 μM (7.5 mg/mL) hIgG and a second incubation (10 min, 25 °C (298 K). A brief spin (15,000 rpm, 10 °C (283 K)) led to pellet formation. Supernatant composition was determined by mixing 30 μL of the supernatant with 20 μL of a sample buffer containing β-mercaptoethanol. Pellet composition was determined by first dissolving the pellet in 50 μL of the same reducing buffer, followed by mixing a 30 μL aliquot of the dissolved pellet with 20 μL of fresh sample buffer. Identical 15 μL aliquots representing the supernatant and pellet composition were loaded on the gel. Control samples contained identical amounts of only (His)_6_-(Z)_2_-(His)_6_, or hIgG. **(ii).\** The same procedure was followed for the hIgG Fc-domain as for intact hIgG, except that partial hIgG digestion was initially accomplished with the FragIT (Genovis) kit according to the manufacturer’s protocol using 0.5 mg/mL hIgG, 20 mM NaCl, and 10 mM NaPi (pH 7.4) in a total volume of 100 μL. Following 3 h of incubation at 37 °C (310 K), centrifugation (15,000 rpm, 2 min, Eppendorf Microfuge 5424-R) was applied, and the sample was either used immediately or stored at −20 °C (253 K).

#### 5.2.7 Binding of FITC-Labeled IgG Antibody to [(His)_6_-(Z)_2_-(His)_6_ :Zn^2+^] Conjugated Biopolymers

For binding studies, [IgG: (His)_6_-(Z)_2_-(His)_6_ domain: Zn^2+^] at a 1:10:13 molar ratio was prepared in PBS. One μL of the commercial antihuman IgG FITC-labeled antibody (2 mg/mL, 80 μM), 2 μL of the (His)_6_-(Z)_2_-(His)_6_ (6.3 mg/mL, 400 μM), and 10 μL of ZnCl_2_ (100 μM) were added to a total volume of 20 μL and incubated at 25 °C (298 K) for 10 min (or overnight at 8 °C (281 K)) prior to imaging with the Olympus IX70 microscope. For the latter, 6 μL of PBS, 2 μL of the commercial antihuman IgG FITC-labeled antibody (2 mg/mL, 80 μM), 2 μL of the (His)_6_-(Z)_2_-(His)_6_ (6.3 mg/mL, 400 μM), and 10 μL of ZnCl_2_ (100 μM) were added to a total volume of 20 μL and incubated at 25 °C (298 K) for 10 min or overnight at 8 °C (281 K), prior to imaging with the Olympus IX70 microscope equipped with the appropriate laser lines. The FITC-IgG peak absorption is at 490 nm; peak emission is at 525 nm.

## Abbreviations

CD: circular dichroism
2D: two-dimensional
FITC: fluorescein isothiocyanate
IgG: immunoglobulin G
hIgG: human immunoglobulin G
SDS: sodium dodecyl sulfate
SDS-PAGE: sodium dodecyl sulfate polyacrylamide gel electrophoresis
STEM: scanning transmission electron microscopy
Native-PAGE: native polyacrylamide gel electrophoresis
(His)_6_-(Z)_2_-(His)_6_ domain: recombinant tandem Z-domains derived from Protein A containing a (His)_6_-tag at their N- and C- termini
